# Role of Minimal (Measurable) Residual Disease Assessment in Older Patients with Acute Myeloid Leukemia

**DOI:** 10.3390/cancers10070215

**Published:** 2018-06-26

**Authors:** Francesco Buccisano, Richard Dillon, Sylvie D. Freeman, Adriano Venditti

**Affiliations:** 1 Hematology, Department of Biomedicine and Prevention, Tor Vergata University of Rome, 00133 Rome, Italy; adriano.venditti@uniroma2.it; 2 Department of Medical and Molecular Genetics, King’s College, London SE1 9RT, UK; richard.dillon@kcl.ac.uk; 3 Institute of Immunology and Immunotherapy, University of Birmingham, Birmingham B15 2TT, UK; S.Freeman@bham.ac.uk

**Keywords:** older AML, multiparametric flow-cytometry, RT-qPCR

## Abstract

Minimal (or measurable) residual (MRD) disease provides a biomarker of response quality for which there is robust validation in the context of modern intensive treatment for younger patients with Acute Myeloid Leukemia (AML). Nevertheless, it remains a relatively unexplored area in older patients with AML. The lack of progress in this field can be attributed to two main reasons. First, physicians have a general reluctance to submitting older adults to intensive chemotherapy due to their frailty and to the unfavourable biological disease profile predicting a poor outcome following conventional chemotherapy. Second, with the increasing use of low-intensity therapies (i.e., hypomethylating agents) differing from conventional drugs in mechanism of action and dynamics of response, there has been concomitant skepticism that these schedules can produce deep hematological responses. Furthermore, age dependent differences in disease biology also contribute to uncertainty on the prognostic/predictive impact in older adults of certain genetic abnormalities including those validated for MRD monitoring in younger patients. This review examines the evidence for the role of MRD as a prognosticator in older AML, together with the possible pitfalls of MRD evaluation in older age.

## 1. Introduction

Incidence of acute myeloid leukemia (AML) is highest among older adults, with a median age of 67 years at presentation [1]. Besides the overall aging of the population, increasing exposure to environmental toxic compounds and the ever more successful use of chemo-radiotherapy in treating other cancers are additional factors leading to the occurrence of so called “secondary” AML [2]. The general outcome of AML in older adults (e.g., ≥60 years old) is poor as compared to younger patients, with less of 20% of patients becoming long-term survivors.

Several reasons explain such a disappointing clinical outcome. First, the genetic profile of elderly AML is often unfavourable due to the higher incidence of poor-risk karyotypes and the lower frequency of good-risk molecular features [3]. Older patients are also more likely to have comorbidities, which complicate the treatment decision-making process and exaggerate the side-effects of therapy [4]. Finally, even for those who tolerate intensive induction chemotherapy and who achieve a morphological complete remission (mCR), relapse rates remain frustratingly high [5].

In light of this, it is critical not only to develop more effective frontline therapies but also to identify appropriate biomarkers that can inform treatment choice and likelihood of success. When it comes to AML, availability of biomarkers with these characteristics is still an unmet need, especially for older adults.

In the modern era, identification of biomarkers represents a fundamental step in the attempt to abandon empirical therapy in favour of risk-driven approaches [6]. Indeed, biomarker-based assessment not only allows individual patients to be directed to a particular treatment, but also permits measurement of the effectiveness of that specific therapy. Biomarkers can be determined at diagnosis (for example genetic and molecular abnormalities), or after treatment delivery. In this context, minimal (or measurable) residual disease (MRD) promises to be a valuable biomarker to investigate the quality of response, and to help guide post-remission decisions.

The role of MRD in elderly AML has been poorly investigated, at least in part due to the reluctance of physicians to treat elderly patients with intensive chemotherapy regimens, together with the renewed interest in low intensity therapy, thanks to the availability of hypomethylating agents (HMAs) [7].

However, more recently the older AML landscape appears to be changing. Careful selection of candidates for intensive chemotherapy and the increasing use of allogeneic transplantation (ASCT) with reduced-intensity conditioning [8] have increased the possibility of achieving a MRD negative mCR even for older patients. In addition, new low-intensity approaches (i.e., HMAs or low-dose cytarabine plus bcl-2 inhibitors) have substantially increased the chances of achieving mCR, some of which have been demonstrated to be MRD negative [9,10,11]. These observations have boosted interest in the role of biomarkers of response quality in elderly patients with AML, paving the way for clinical trials incorporating MRD-based risk stratification.

The use of an MRD-driven treatment allocation in younger AML patients might allow physicians to deliver an intensity of treatment proportional to the level of chemosensitivity of each patient, possibly sparing excessive toxicity (i.e., ASCT) for patients with a MRD-negative mCR [12]. The aim of this review is to explore the clinical evidence supporting the role and value of MRD monitoring in older patients with AML.

## 2. Flow Cytometric MRD

**Figure 1:**
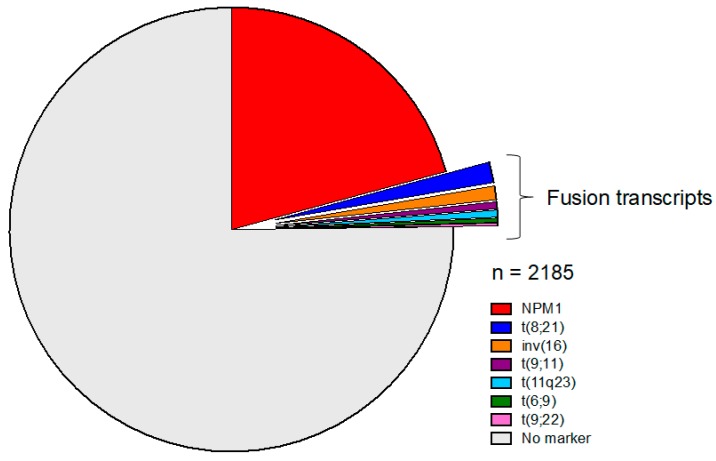
Prevalence and distribution of standardised molecular Minimal Residual Disease (MRD) targets in adults >65 years entering the National Cancer Research Institute (NCRI) Acute Myeloid Leukemia (AML)-16 trial [47].

Multiparametric flow cytometry (MFC) is a single cell immunophenotypic approach that requires only a few hours of laboratory processing time from sample receipt to result, in order to assay several million hematopoietic cells for MRD detection in a patient’s bone marrow (BM) or peripheral blood (PB) sample. Leukemic genetic and epigenetic abnormalities may dysregulate cell expression of molecules compared to normal hematopoietic maturation, thus generating a range of aberrant immunophenotypic profiles (usually referred to as leukemia-associated immunophenotypes/LAIPs) that are the MFC detection targets. By integrating flow cytometric information on relative cell size, granularity and multiple cell markers detected simultaneously by fluorochrome-tagged monoclonal antibody combinations, cells with LAIPs can be discriminated from their normal hematopoietic counterparts. LAIPs can be defined in diagnostic samples and then tracked but may also be detected in follow-up samples independently of diagnostic immunophenotypic data by screening for blast aberrancies that are sufficiently “different-to-normal” (i.e., different to reference control marrow profiles). LAIPs can be characterized in ~90% of diagnostic samples from older [13] as well as younger patients while reverse transcription polymerase chain reaction (RT-qPCR) MRD targets (fusion genes, NPM1 mutations) are present in only ~20–30% of adults over 60 years old compared to ~60% of younger adults (see below and Figure 1) [14]. However, for some patients a suitable LAIP target constitutes only a relatively minor subpopulation of the total leukemic blasts at diagnosis. In these cases, the typical LAIP sensitivity range of 10^−3^–10^−4^ can be maintained by evaluating sufficient cells in the follow-up when BM samples are adequate.

In addition to flow cytometric MRD detection being applicable to most older patients, this methodology provides other relative advantages when considering the specific context of elderly AML. Aging is associated with an increased prevalence of clonal hematopoiesis contributing to the accumulation of mutations in mature cells [15,16]. Since mature cells are not leukemia-initiating, this presents pitfalls for the clinical interpretation of genetic MRD assays in older patients and may be a particular issue for MRD monitoring during targeted therapy induced maturation [17] as routine MRD assays are performed on whole BM. Unlike FISH, PCR or NGS, immunophenotyping by flow cytometry allows the evaluation of MRD in the hematopoietic compartments most relevant for predicting relapse (i.e., blasts) without requiring cell separation to exclude mature cells. Additionally, it can provide an estimate of BM hemodilution to assess which samples are suboptimal.

The increasingly routine use of 8–10 fluorochrome-tagged antibodies per tube of cell suspension allows both better identification of myeloid blast subpopulations as well as screening for aberrancies. However, when designing flow cytometric AML MRD panels, inclusion of increasing numbers of antibodies to allow detection of rarer useful LAIPs has to be counterbalanced with cost, cell requirements, and technical fluorescent compensation challenges. Recently, antibody mixtures have been devised that combine several markers of aberrancy on one fluorescent channel and thus reduce the number of separate LAIP panel tubes required per sample. Although such an approach loses the information gained from analyzing single markers (when assessing LAIP specificity in heterogeneous blast populations or identifying potential immunotherapy targets), it is informative and sensitive for detecting leukemic cells when applied to CD34+CD38− stem/progenitor cells [18]. The merged immunophenotypic aberrancy from the combined markers was shown to be absent or extremely rare (<10^−4 to −5^) on the normal hematopoietic counterpart, even during post chemotherapy regeneration.

As reviewed recently [19,20,21,22,23,24], AML MRD results from flow cytometric measurement have to be interpreted in the context of both methodological and sample limitations. Some of these are especially relevant to the older age cohort. Sensitivity of all MRD assays is comprised by suboptimal samples. Hypoplastic regeneration from poor hematopoietic reserve in older patients may increase the number of MRD-unassessable samples. Perhaps surprisingly, the frequencies of inadequate bone marrow samples sent after first induction were comparable in the NCRI AML16 trial for older patients (9.8% of 744 samples) and NCRI AML17 trial for younger patients (7.9% of 1906 samples) [25].

A further consideration for reporting flow cytometric MRD in older patients due to the likelihood of background dysregulated clonal hematopoiesis are the implications of aberrant immunophenotypes associated with myelodysplasia. Cross-lineage expression of lymphoid markers such as CD7 and CD56 on myeloblasts are present in up to 30% of AML patients at diagnosis and are commonly selected LAIPs for AML MRD monitoring. These aberrancies are also observed on blasts in a similar percentage of low-risk myelodysplastic patients [26,27,28,29,30,31] (Table 1) and therefore not specific for AML.

Moreover, in MDS, as in AML, immunophenotypic aberrancies of CD34+CD38− putative leukemic stem cells (LSC) also occur and may be prognostic for progression to AML [35]. Although CD7 and CD56 cross-lineage expressions are associated with certain leukemic genetic subtypes [36] there is as yet no data linking these immunophenotypes to any myelodysplasia mutation combinations. It is possible that MRD positivity by a pre-leukemic aberrant immunophenotype measures residual leukemic blasts even if the aberrancy was acquired earlier in evolution to AML. Studies combining flow cytometric monitoring and mutation profiling may clarify this and allow a more accurate assessment of relapse risk in elderly AML with antecedent MDS.

### Clinical Significance of Flow Cytometric MRD in Older Adults

Most data on the prognostic relevance of MRD in older AML patients, including the two studies of intensively treated adults >60 years, have been obtained by MFC quantification of MRD [13,37]. The results of the NCRI-AML16 study [13] demonstrated that, using intensive chemotherapy, a MRD negative mCR is inducible in 51% of patients after cycle 1 (C1) and in 64% of patients after cycle 2 (C2), respectively. Patients were considered MRD positive if detected levels of residual leukemic cells were higher than the MFC assay sensitivity threshold (0.05–0.1% for most LAIPs). When MRD negativity was achieved, patients had a better 3-year survival (C1: 42% vs. 26% in MRD-positive patients, *p* < 0.001; C2: 38% vs. 18%, respectively; *p* < 0.001) and reduced cumulative incidence of relapse (C1: 71% vs. 83% in MRD-positive patients, *p* < 0.001; C2: 79% vs. 91%, respectively; *p* < 0.001). In a smaller series [37], where the MRD status of 81 older patients was compared in terms of clinical outcome to a population of younger AML patients, MRD-negative mCR was confirmed to be a favorable biomarker despite the frequency of MRD negativity being lower than a younger population (11% and 28%, respectively, *p* = 0.009). MRD negativity resulted in longer 5-year disease-free survival both in older (57% vs. 13%, *p* = 0.0197) and younger patients (56% vs. 31%, *p* = 0.0017). As a further observation, despite the selection of a threshold that was lower than in the NCRI-AML16 trial (0.035% vs. 0.05–0.1%), CIR of MRD-negative patients was double that in the MRD-negative younger cohort (42% vs. 24%, respectively). This is in line with the NCRI report, where 3-year CIR remained remarkably high for older patients achieving MRD negativity, although it is notable that their median time to relapse was significantly longer than the MRD-positive group (17.1 months compared to 8.5 months by C1 MRD status).

Another relevant field of MRD application is in post-treatment surveillance and early relapse identification [38]. This is an underexplored area in older AML, due to the paucity of patients submitted to a treatment with the aim of achieving a mCR. The increasing success of HMA-based protocols that differ from conventional chemotherapy in their mechanisms of action and dynamics of response has contributed to a general scepticism in applying MRD monitoring in this setting [39]. The clinical relevance of MRD monitoring in older patients with AML treated with HMAs alone has been recently explored [11]. A group of 116 patients were monitored by high-sensitivity 8-color MFC for MRD achievement during therapy with azacytidine, decitabine and guadecitabine. A significant advantage of MRD-negative patients in terms of cumulative incidence of relapse was observed (83% vs. 43%, *p* < 0.001); however, this did not translate into improved survival. Nevertheless, this experience determines the proof of principle that a good quality mCR may be achieved also by HMA therapy, and that MRD is a suitable biomarker also in this setting. Furthermore, addition of novel targeted agents to HMAs in ongoing clinical trials has led to higher rates of mCR that are achieved more rapidly than with HMAs alone and has raised interest in the ability of such combinations to eradicate MRD [9,10]. In the future, this issue may be more properly addressed by MRD monitoring in prospective trials. In this view, the EORTC/GIMEMA AML1301 trial (trial gov. identifier no. NCT02172872) is prospectively evaluating, in parallel, the impact on MRD levels of a classical 3 + 7 versus decitabine given over the extended schedule of 10 days. Results of this trial are eagerly expected since the 10-day decitabine schedule might prove to be the new standard of care for older patients and to induce excellent quality responses. Assays monitoring candidate LSC-containing cell populations could also have a role in assessing depth of remission in this context. Reduction of candidate LSC-containing cell populations tracked by flow cytometry was observed in responders to azacytidine in a cohort that included AML as well as MDS patients [40].

## 3. Molecular MRD

Molecular techniques for assessment of MRD rely on the detection of leukaemia-specific genomic abnormalities in nucleic acid preparations derived from PB or BM samples. In general, molecular techniques provide significantly greater sensitivity and specificity compared to MFC-based assays; however, they are not yet applicable to all patients, and in particular, there are no validated molecular MRD markers for a majority of older adults [14]. Furthermore, the majority of studies of the prognostic importance of molecular MRD status have been performed in younger adults, so the predictive MRD thresholds defined therein cannot necessarily be applied to older patients. Finally, molecular MRD monitoring is not currently useful for patients who are treated non-intensively.

The exquisite sensitivity of some molecular MRD assays allows extension of their scope beyond the early time points typically employed for MFC assessment. Achievement of a negative molecular MRD status (“molecular complete remission”, “CR_MRD−_”) at the end of treatment has begun to gain acceptance therapeutic objective and pre-requisite for cure, implying that treatment intensity should be adapted to ensure this is achieved [41,42]. Secondly, by virtue of their high sensitivity, molecular MRD assays may be deployed in surveillance for disease relapse after the completion of treatment; re-emergence of the relevant molecular marker (“molecular relapse”) may be detected in advance of morphological relapse, allowing pre-emptive therapeutic intervention and potentially improving long-term outcome [42].

It is important to note that these two principles of molecularly guided treatment were pioneered in and are now established as standard of care for acute promyelocytic leukaemia (APL), where, coupled with the availability of effective salvage therapies (namely arsenic trioxide and gemtuzumab ozogamycin), they have led to a dramatic reduction in the risk of haematological relapse [41,43]. Increasing availability of novel and effective therapeutic agents for AML (e.g., venetoclax) [44], and expanding access to ASCT for patients >65 are likely to significantly increase the number of patients treated with curative intent; however, these therapies are accompanied by very significant toxicity and economic costs. Molecularly-guided therapy has the potential to personally tailor the treatment of AML in older people, allowing such therapies to be deployed only for patients who will benefit from them.

### 3.1. Current Methods for Molecular MRD Detection

The most established method for molecular MRD assessment is RT-qPCR. In this technique, up to 20 mcg RNA extracted from patient samples (equivalent to ~1 million cells) is reverse transcribed (RT) into complementary DNA (cDNA) and subsequently amplified by quantitative PCR, allowing precise measurement of the molecular target. RT-qPCR is ideally suited to the detection of fusion transcripts generated from in-frame somatic rearrangements [45] and insertion/deletion (InDel) mutations, particularly those in exon 12 of *NPM1* [46]. Assays for these targets produce almost no background amplification, providing excellent specificity. Sensitivity is generally in the range of 1:10^4^ to 1:10^6^ and depends on a number of factors, especially the level of expression of the target fusion or mutant transcript and the quality and quantity of RNA generated, which is in turn dependent on the age of the sample.

Figure 1 shows the proportion of older adults (>60 years) with a molecular lesion suitable for monitoring by RT-qPCR entering the AML16 study, which enrolled 2783 patients. Overall, only 4.2% of patients with an evaluable karyotype (92/2185) had a recognised gene fusion, of which the most frequent were the core-binding factor translocations t(8;21)(q22;q22)/*RUNX1-RUNX1T1* (1.5%) and inv(16)(p13q22)/*CBFB/MYH11* (1%). In contrast, 21% of all older adults and 38% of those with a normal karyotype carried *NPM1* exon 12 InDel mutations [47].

### 3.2. NPM1 Mutated AML

InDel mutations in exon 12 of *NPM1* represent near-ideal molecular MRD targets. They are leukaemia-specific and are never found in clonal haematopoiesis [48]; they reliably track the leukemic clone and are concordant between diagnosis and relapse in >90% of patients [49,50]; they are highly expressed, permitting very high assay sensitivity [46]. Multiple studies have confirmed the prognostic implications of *NPM1* MRD status in younger patients [49,51]. The UK AML17 study showed that PB status after two cycles of chemotherapy was highly predictive of outcome in patients aged <69. Three-year overall survival (OS) was 24% in patients who were MRD-positive at that time point compared with 75% for those who tested negative. In multivariate analysis with clinical parameters and mutational profile, MRD status was the only factor to retain significance. These results are supported by the French ALFA0702 study, which enrolled patients aged <60 years, and showed PB MRD negativity or a >4 log reduction in transcript levels after one cycle of therapy was associated with a 3-year OS of 91%. The German-Austrian AMLSG study similarly found that bone marrow MRD negativity after two cycles of chemotherapy in patients aged <60 years was associated with a 4-year OS of 82% [52].

**Figure 2:**
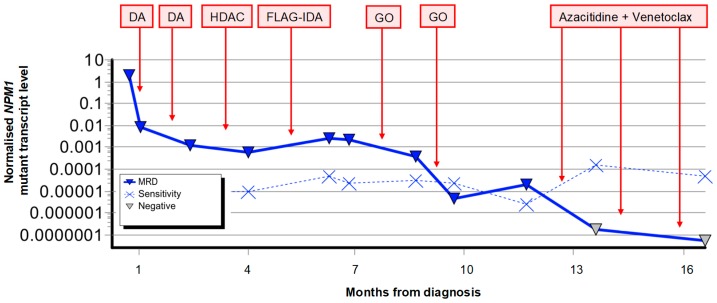
Example of MRD-guided therapy to achieve durable molecular complete remission in a 72-year old male ineligible for stem cell transplantation due to renal impairment [58]. DA: daunorubicin and cytarabine. HDAC: high dose cytarabine. FLAG-IDA: fludarabine, cytarabine, granulocyte colony stimulating factor and idarubicin. GO: gemtuzumab ozogamycin.

There are two major precautions when seeking to apply *NPM1* MRD monitoring to patients older than 65 years. First, although *NPM1* mutant AML is associated with a generally favourable rate of overall survival in younger adults, particularly in the absence of a *FLT3* internal tandem duplication (ITD) [53,54], this does not hold true in older patients. While the chance of achieving complete remission with induction therapy remains relatively high (60–80%) [55,56] the chance of relapse is markedly higher in older *NPM1* mutant patients (1-year CIR 71% in patients aged >65 compared with 35% in those aged 55–65) and the 2-year overall survival significantly lower (19% and 39% respectively) [55]. Since the most predictive early time points and thresholds appears to vary even between cohorts of younger adults, these cannot easily be applied to older patients without further study. Notwithstanding this, attainment and maintenance of a molecular CR are pre-requisites for long-term cure in any age group, and *NPM1* monitoring may be useful both to personalise treatment to ensure this is achieved (as demonstrated in Figure 2) and for the early detection of relapse, which is particularly pertinent in this age group with limited capacity to tolerate intensive salvage chemotherapy. The second caveat relates to the small number of cases where relapse occurs from an ancestral clone which is *NPM1*-negative. Although this has been clearly documented [57], it is a rare phenomenon in younger adults where <1% of relapses are *NPM1* wild-type [49]. Prevalence of this phenomenon in older adults is unknown, but is likely to be higher due to the increased proportion of cases evolving from pre-existing non-malignant clonal haematopoiesis as discussed below.

### 3.3. Core Binding Factor (CBF) AML

The second major group of patients with well-established molecular targets for MRD monitoring are those with CBF AML defined by the presence of either the inv(16)(p13q22) *CBFB-MYH11* or the t(8;21)(q22;q22) *RUNX1-RUNX1T1* fusion. Although associated with a high chance of long-term cure in children and young adults, older patients with CBF AML have a significantly worse prognosis due mainly to a high incidence of relapse (~60%) [3,59]. Published studies regarding the impact of MRD on outcome have included few or no patients >60 years [60,61,62,63], and the significant difference in relapse risk indicates that thresholds and checkpoints defined in younger adults cannot simply be transposed onto older adults without further validation in this population. Nevertheless, sequential MRD monitoring during morphological remission is recommended [41] as this can identify patients at high risk of imminent relapse for pre-emptive intervention. Serial MRD measurement may also be used to guide consolidation or maintenance therapies such as HMAs [11,64] and may be increasingly useful as the range of accessible novel therapeutic agents expands.

### 3.4. Emerging Molecular MRD Techniques

Since the majority of older patients do not have an established molecular MRD marker, novel platforms other than RT-qPCR are of great interest. The emerging techniques with greatest promise in this regard are droplet digital PCR (ddPCR) and next-generation sequencing (NGS), a number of recent studies have highlighted both their potential utility and limitations. A general principle emerging from this work relates to clonal heterogeneity; distinct clones within an individual may show marked differences in their response to therapy and in their potential to generate relapse. Unlike gene fusions and mutations in *NPM1*, which are largely leukaemia-specific and are stable between diagnosis and relapse, mutations in *DNMT3A*, *ASXL1*, and *TET2* often persist at a high level in patients in long-term mCR, suggesting a reversion to pre-existing non-malignant clonal haematopoiesis of indeterminate potential (CHIP), indicating these mutations are not sufficiently leukaemia-specific to serve as MRD targets. This is highly pertinent to older adults given the strong relationship between CHIP and age [16,48,65,66,67,68]. In contrast, mutations in genes such as *FLT3* and *NRAS* are frequently lost or gained at relapse; their lack of stability indicates they cannot provide sufficient sensitivity or reliability for MRD monitoring. Many investigators have sought to bypass these limitations by tracking multiple mutations concurrently, an approach which appears to enhance robustness and at the same time provides greater insights into the issues outlined here, which will in turn allow further refinement of these techniques. RT-qPCR is generally unsuited to the sensitive detection of single nucleotide variants (SNV) due to background amplification of the wild-type sequence. In ddPCR, the sample is partitioned into thousands of oil-bound droplets, each acting as an independent reaction. The droplets are then read as discreet positive or negative events allowing non-specific amplification to be largely excluded and quantitative precision to be improved [69]. A recent study of 72 patients (median age 62 years) used ddPCR to track mutations found at diagnosis either by whole exome or targeted sequencing [70]. Overall, 90% of patients had a detectable mutation in a bone marrow sample taken in complete remission, at levels between 0.002% and 46.2%. Mutations in *DNMT3A* frequently persisted at a high level and their frequencies often did not correlate with those of other mutations tracked in the same sample, a phenomenon that was also noted for other genes such as *ASXL1*, *TET2*, *IDH1*, *RUNX1*, and *WT1*. To overcome this, two analyses were performed. First for the lowest frequency mutation present in each sample, a threshold of 0.01% separated patients into two groups with 5-year OS of 19% and 50%. Second, for the highest frequency mutation excluding *DNMT3A* a cut-off of 0.1% identified two groups with 5-year OS of 16% and 65% respectively.

EC-NGS provides a potentially less cumbersome approach to track multiple mutations without needing to design an individual assay for each. This is particularly relevant to genes where mutations may occur at numerous sites rather than in “hot spots” such as *RUNX1*. A study of 103 patients (median age 69 years) with *RUNX1* mutated AML used PCR amplification followed by amplicon sequencing on the Roche 454 platform [56] affording sensitivity of up to 1:800. A threshold of 3.6% was informative with respect to outcome: the 74% of patients below this threshold after one cycle of induction therapy had a median event-free survival (EFS) of 21 months compared with 5.7 months for the remaining 26% of patients. This approach was extended to track a total of 564 variants in CR bone marrow samples from 50 patients (median age 50.8 years) [71]. Complete clearance of all mutations below a level of 2.5% was associated with a median EFS of 17.9 months compared with 6 months in those patients who had at least one mutation detectable above this threshold.

A major drawback of NGS techniques for MRD assessment is the background error rate which results from imprecision in both DNA polymerase activity and optical detection and varies markedly across the genome, and until recently, has limited the impact of NGS in this field. One way to mitigate this is to perform statistical analysis to determine the background error rate at each base position interrogated; this was applied in a study involving 482 adults <65 years [72] and resulted in a maximum sensitivity of 0.1% for loci with the lowest background error rates. After exclusion of mutations in *DNMT3A*, *TET2*, and *ASXL1* (“DTA”, which did not correlate with relapse), detection of any mutation in CR was associated with a 5-year CIR of 58.3% compared with 33.9% in patients with no mutation detected. Presence of a non-DTA mutation in CR retained powerful independent prognostic significance in multivariate analysis and provided additional prognostic power when combined with FCM-MRD. A second and potentially more robust method for application of NGS to MRD analysis is to incorporate novel error correction (EC) techniques, which have the potential to reduce background error rates significantly and can increase sensitivity by at least one order of magnitude [73]. In EC-NGS, each DNA fragment is ligated to a random 8–12 base pair oligonucleotide (often referred to as a molecular barcode or unique molecular identifier, UMI). The prepared library is later amplified by PCR, and multiple fragments deriving from the same parent DNA molecule are sequenced independently, after which a bioinformatic algorithm is applied to remove variants, which are likely artefacts, not present in each copy. Using this approach in a study of 69 patients, sensitivity of at least 0.2% could be achieved for most loci [74]. While persistence of one mutation in CR was frequent (affecting *DNMT3A*, *U2AF1*, *TET2* and *SRSF2*), associated with older age, and did not affect outcome, the presence of two or more mutations was associated with a 2-year leukaemia-free survival of 19.8% compared with 64.9% for those with one or no mutations detected. Using EC-NGS, it is clear that patients treated non-intensively retain significant levels of MRD despite appearing to be in complete remission using non-corrected NGS approaches [75]. It remains unclear how useful such techniques will be for patients who are not treated with curative intent, given the lack of relationship between clinically important variables (blast clearance, transfusion independence) and MRD status [75,76]. This and many other important questions in the field of molecular MRD in older patients will hopefully be addressed by large prospective studies over the next decade.

## 4. Conclusions

Despite the powerful and robust prognostic value of MRD, demonstrated across multiple independent platforms, laboratories, time points, and trial protocols [19,24,77], its application to older adults remains underexplored. There are two likely explanations for the lack of data highlighted in this review. First, older patients with AML are less likely to be enrolled in studies involving intensive therapy [5]. Second, since the increasingly popular low-intensity therapies (i.e., HMAs [9,10,11]) are able to prolong survival even without the achievement of a mCR, there has been general scepticism about the relevance of MRD studies in this context, and many studies involving these agents have not collected MRD data [39]. Improvements in supportive care and the availability of ASCT up to and beyond the age of 70 years has significantly extended the use of intensive regimens for older adults; moreover, the demonstration that a high quality, MRD-negative mCR may be realistically pursued in low-intensity therapy (including potentially in combination with Bcl2 inhibitors) [10] has prompted new interest in MRD-monitoring for these therapies, at least by flow cytometry.

Due to the lack of evidence we highlight, coupled with clear age-dependent differences in disease biology and outcome, the prognostic significance of MRD status remains largely unknown in older adults and requires further study. In the two MFC-based studies, older patients achieving a MRD-negative mCR still had a substantial risk of relapse and outcomes remained less favourable, regardless of the post-remission treatment received [13,37]. This likely reflects differences in disease biology between younger and older patients, including but not limited to an increased proportion of cases evolving from (and perhaps reverting to) MDS and a higher frequency of oligoclonal leukemic/preleukemic populations with different treatment sensitivities and relapse kinetics [78]. Molecular markers such as *NPM1* are both less frequent in elderly AML, and do not appear to carry the same prognostic implications compared with younger adults due mainly to a significantly elevated overall relapse risk in all MRD-defined groups [55]. A second factor contributing to this phenomenon in a subgroup of patients is relapse from an ancestral clone which is *NPM1* negative; in elderly AML, where a pre-existing clonal hematopoiesis is often documented despite mCR achievement, this phenomenon may be even more relevant. Similarly, persistence of age-related clonal hematopoiesis mutations as detected in CHIP may hamper the detection of MRD using NGS-based technologies in this age group [72,79].

Despite these observations, the powerful prognostic value of MRD is likely to prove useful in managing older adults with AML. As an accurate measure of chemosensitivity, it may prompt physicians to continue treatment or design specific consolidation or maintenance approaches aimed at achieving and maintaining an MRD-negative mCR whilst minimizing treatment toxicity. This differs from the aim of treatment de-escalation including avoidance of ASCT in younger patients with a MRD-negative mCR [12].

Since MRD negativity following both standard chemotherapy and HMAs is associated with better outcome, there is a rationale for applying MRD results to select patients likely to benefit from investigational therapies in future trials. Potentially, MRD response could also be used as an early surrogate outcome endpoint. In the intensive NCRI AML16 study, there was a trend for improved survival from the addition of a consolidation cycle only for the MRD negative patients; the same addition was detrimental to MRD positive patients with a significantly inferior survival. This supports the hypothesis that MRD negativity in older adults identifies a subgroup with more chemosensitive disease, suitable for further treatment consolidation, whereas the disappointing long-term outcome of MRD-positive patients is not altered by additional similar chemotherapy [80]. These poor responders may, however, benefit from clinical trials investigating novel therapies, possibly augmenting the quality of remission and the long-term antileukemic effect of ASCT. Ongoing studies such as the NCRI AML18 trial are testing MRD risk-directed therapy for older adults. It as yet unknown whether reducing MRD to lower levels translates into improved median survival and/or decreased requirements for supportive measures such as transfusions, so this also requires investigation, particularly when evaluating novel therapies.

In conclusion, MRD as assessed by MFC or molecular techniques (RT-qPCR, NGS) represents a powerful biomarker that is able to distinguish subgroups of patients with dramatically differing prognosis. Although the biological uniqueness of elderly AML leads to additional considerations (particularly clonal mutation persistence and immunophenotypic aberrancies) [24], its role in improving clinical decision-making, optimising outcome and targeting novel therapeutic agents for older patients with AML is promising and clearly warrants further evaluation in carefully designed prospective studies focused on this steadily increasing population.

## Figures and Tables

**Table 1:** Frequency of aberrant cross-lineage expression on myeloblasts in MDS versus AML.

Immunophenotypic Myeloid Blast Aberrancy	Low/Int-1 Risk MDS Patients with Aberrancy (%) [26,27,31]	High Risk MDS Patients with Aberrancy (%) [27,31]	AML > 60 Years * Patients with Aberrancy at Diagnosis (%) [13]	AML < 65 Years * Patients with Aberrancy at Diagnosis (%) [32,33,34]
CD7 cross lineage expression	3.5–22%	3.5–16.7%	23%	25–32%
CD56 cross lineage expression	3.3–18%	?	19%	15–21%
CD5 cross lineage expression	<2%	<1%	ND	<1%

* Patients for standard/intensive chemotherapy.
